# The Institutional Library

**Published:** 1913-11-01

**Authors:** 


### THE INSTITUTIONAL LIBRARY.

### A School Clinic Handbook.

School Clinics at Home and Abroad. By Lewis D. Cruickshank, M.D., D.P.H. Illustrated. (London: The National League for Physical Education and Improvement. 1913. Price 2s. 6d.)

School clinics are surely, if slowly, establishing for themselves a permanent place in the new order of educational affairs. In 1908 the inspection of school children was started all over the country, and it was inevitable that the mass of defects which then began to be discovered should be followed by some demands for their cure, amelioration, and, above all, prevention. Medical officers, teachers, and secretaries of education authorities are familiar with the struggles, not to say squabbles, associated with the endeavours to get the children treated. To obey the ambiguous dictum of the Board of Education—"to make use of existing institutions"—has been beset with difficulties which only those actually dealing with the children can fully appreciate. The question, therefore, of the establishment of school clinics is still a very live one, and by every section of institutional workers is one on which full information is required. This book has been specially prepared for this purpose. It gives the result of an investigation conducted by the National League for Physical Education and Improvement. The bulk of the text is by Dr. Lewis Cruickshank, who is principal of the College of Hygiene and Physical Training at Dunfermline, and who has taken an active part in the organising of clinics. The volume contains a wealth of information of a kind that will enable the medical officer, hospital officer, and, say, the county councillor alike to appreciate the difficulties which have been overcome or are yet to be met in organising the treatment of the children found to be disabled in varying degrees for their coming struggle for existence. This book also gives the right point of view for understanding the significance of the official reports of local medical officers and the numerous Blue-books and memoranda issued by the Government. It states the case for the establishment of school clinics in an unbiassed fashion; an important section discusses the advantages and disadvantages of the several methods which are impartially put forward of dealing with the many details of organisation. Above all, the book bristles with *practical* detail. The object of the League is not so much to discuss scientific ideas as to promote their application in practice. This is why the present volume combines a minimum of theory with a maximum of practical detail. The utilising of existing voluntary agencies for the treatment of the diseases of school children has been fully tested in most of the large educational centres; but, although these have accomplished a deal of excellent work, it is generally agreed that they are unsuitable to cope with the great numbers of children requiring attention. A new institution has, therefore, become necessary, and the fullest information is available in this book on the organisation and administration of school clinics—an institution which grows naturally out of the school organisations and which is admirably fitted to meet the special needs of the case. Here is not the place to argue the advantages of school clinics, but it may be permissible to draw attention to one point which is not, perhaps, sufficiently appreciated, and that is the way in which the clinic facilitates control and regularity of attendance. The "following up" of cases is infinitely more efficient when treatment is given in a clinic than in a busy hospital. Over and over again the reports of school medical officers remark on the excessive leakage between inspection and treatment. This excellent volume deserves to be widely known, for it presents a complete survey of the whole question of school clinics—function, organisation, and management of the several special departments, such as the dental, orthopædic, and the ophthalmic. The details of equipment and finance are succinctly given from actual examples. Not the least important is Part III., which deals in a most instructive manner with the school clinics abroad. The book has many excellent photographs, affording object-lessons of great utility, and is prefaced by a general introduction by Dr. W. L. MacKenzie, of the Local Government Board for Scotland.

The Mechanical Treatment of Abdominal Hernia. By W. B. de Garmo, M.D. (Philadelphia and London: J. B. Lippincott Company. Pp. 147. Price 6s. net.)

Modern surgery has brought the radical cure of hernia to such a pitch of perfection that the older means of at least relieving this common condition are apt to be relegated more and more into the background. Nevertheless, even still there are many thousands of people who for various reasons—some good, some bad—do not submit themselves to operative measures, but rely on some form of mechanical support, usually a truss. With the increasing aversion of surgeons to countenance trusses whenever a radical cure seems to be feasible and indicated, the selection and design of trusses for particular cases falls more and more into the hands of instrument-makers; or, not infrequently, into those of quacks and charlatans. Professor de Garmo's book shows what a lot of art there is in the truss treatment of hernia, properly regarded from surgical principles; and his very careful and practical exposition of the subject will certainly be found of immense utility by any practitioner who has the supervision of a case of hernia which for any reason is not submitted to operation.

### Medical Education of the Public.

The Heart and Blood-Vessels. By I. H. Hirschfeld, M.D. (New York and London: Funk and Wagnalls Company. 1913. Pp. 336. Price 5s. net.)

Not long ago in The Hospital we mentioned the ideals of Dr. Soresi, an American doctor who advocates that in everyone's education there should be included a course of systematic instruction in modern medicine and surgery and the symptoms of disease. On that occasion we had under notice a book on popular medical matters by Dr. Woods Hutchinson. Now we take up yet another one of much the same object—namely, the instruction of the general public in questions of health and hygiene. In this case, however, the author, Dr. Hirschfeld, deals with certain special aspects of health and disease, the care of the heart, blood-vessels, and circulatory mechanism in general. Consideration of some of the disorders of this system leads to a discussion on venereal disease and the sexual instincts, about which he does not hesitate to speak his mind very plainly. Many will not agree with all his views, but they are stated temperately and with dignity; and the mere fact of their discussion is a sign of the times. Indeed, his discourse on the heart and blood-vessels is eminently sound and sane, and is in addition written in language commendably free from colloquial Americanisms.

